# The complete chloroplast genome of *Caroxylon passerinum* (Chenopodiaceae), an annual desert plant

**DOI:** 10.1080/23802359.2021.1994891

**Published:** 2022-02-27

**Authors:** Wei Xie, Chi Zhang, Yuguo Wang, Yunfei Zhang

**Affiliations:** a Ministry of Education Key Laboratory for Biodiversity Science and Ecological Engineering, Institute of Biodiversity Science, School of Life Sciences, Fudan University, Shanghai, China; b Natural History Research Centre of Shanghai Natural History Museum, Shanghai Science and Technology Museum, Shanghai, China

**Keywords:** *Caroxylon passerinum*, chloroplast genome, phylogenetic analysis

## Abstract

*Caroxylon passerinum* is an important constructive species, which is widely distributed in both desert and desert steppe in north-western China. *C. passerinum* is one of hosts of holoparasitic *Cistanche* species. In this study, we report the complete chloroplast genome sequence of *C. passerinum*, which is 150,925 bp in length and comprises a large single-copy region (83,057 bp), a small single-copy region (18,180 bp), and a pair of inverted repeats (24,844 bp). It encodes 132 unique genes, including 89 protein-coding genes (PCGs), 35 tRNAs, and eight rRNAs. The overall GC content of this chloroplast genome is 36.8%. Maximum likelihood (ML) phylogenetic tree strongly supports that *C. passerinum* is closely related to the hosts of *Cistanche deserticola*, *Haloxylon persicum* and *Haloxylon ammodendron*.

*Caroxylon passerinum* (Chenopodiaceae) is an annual leaf-succulent and fruiting subshrub with extreme xerophyte and resistance to wind and cold (Akhani et al. 2007). Its populations are fragmentedly distributed in Inner Mongolia, Ningxia, Gansu and Xinjiang, as well as in southern Mongolia and Central Asia at elevations ranging from 1000 to 3000 m (Gao et al. 2009). As one of major constructive species, it is widely distributed in both desert and desert steppe in north-western China, with key ecological functions on sand fixation and preventing desertification (Reed 2003). *C. passerinum* and local medicine plants, the species of *Cistanche* such as *Cistanche salsa*, form a parasitism system (Qin and Liu 2010), which is a good system to study horizontal gene transfer (HGT) (Liu et al. 2020). In order to better understand its genomic structure and organization of *C. passerimum*, and its phylogenetic position in Chenopodiaceae, we sequenced the complete chloroplast genome of *C. passerinum* and compared with those of its relatives. The complete chloroplast genome of *C. passerinum* is the first report for any member of the genus *Caroxylon*.

We collected samples of *C. passerinum* in Zhongwei, Ningxia, China. The voucher specimens were deposited in the herbarium of Fudan University (FUS). Total genomic DNA was extracted from silica-gel dried leaves. Genomic DNA was sequenced using the Illumina Hiseq 2500 (Illumina, San Diego, CA, USA), with 150 bp paired-end (PE) sequencing. The chloroplast genome of *Suaeda glauca* (GenBank: MK867773) (Jian et al. 2019) served as our reference genome. Chloroplast assembling was carried out according to the previous method (Xu et al. 2020). The complete chloroplast genome was assembled by SOAPdenovo2 v2.04 (Luo et al. 2012) and Bowtie2 v2.3.4.1 (Langmead and Salzberg 2012). GapCloser v2.04 (Luo et al. 2012) was used to fill gaps among contigs. This draft genome was annotated using DOGMA (Wyman et al. 2004) and GeSeq (Tillich et al., 2017). To determine the phylogenetic placement of *C. passerinum*, a maximum-likelihood (ML) tree was reconstructed using RAxML v8.2.10 (Stamatakis 2014). tRNA genes were predicted using tRNAscan-SE v1.3.1 (Lowe and Chan 2016).

The chloroplast genome of *C. passerinum* is 150,925 bp in length (GenBank accession number: MW192441). It comprises a large single-copy region (LSC: 83,057 bp), a small single-copy region (SSC: 18,180 bp) and a pair of inverted repeats (IR: 24,844 bp). The overall GC content genome is 36.8%. A total of 132 unique genes were annotated, including 89 protein-coding genes, 35 tRNAs, and eight rRNAs.

To validate the phylogenetic position of *C. passerinum*, 18 species from Chenopodiaceae and three species are from Amaranthaceae as complex outgroup were used to construct a ML phylogenetic tree. The sequences were aligned using MAFFT v7.309 (Katoh and Standley 2013) and the tree was analyzed with RAxML v8.2.11 (Stamatakis 2014). Phylogenetic analysis showed that *C. passerinum* is sister to members of *Haloxylon.* Besides, our results comfirmed that *Caroxylon* and *Haloxylon* had a closer relationship with *Sueada* and *Bienertia* than with *Chenopodium* (Figure 1).

**Figure 1.**
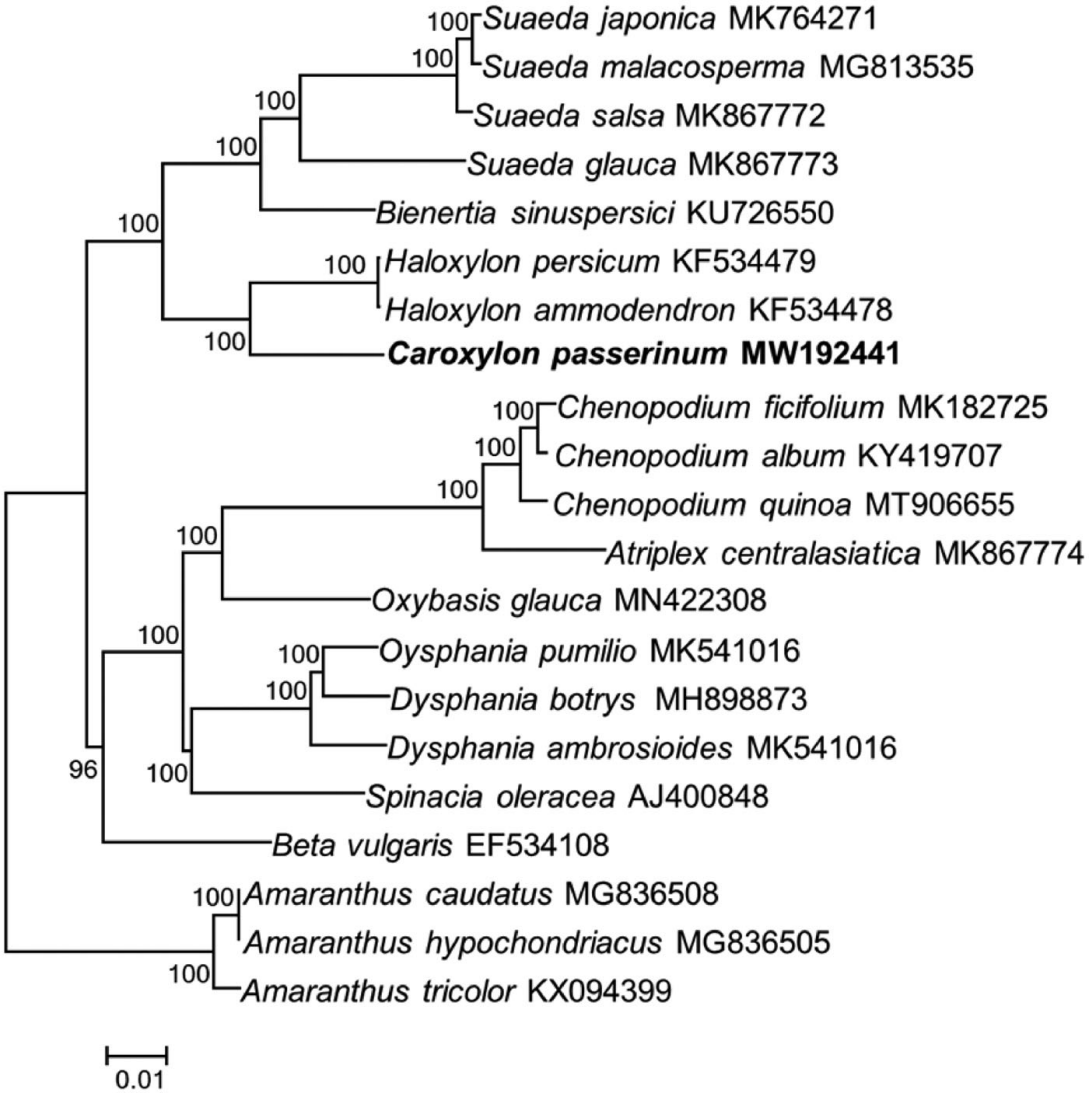
Molecular phylogeny of Chenopodiaceae using chloroplast genomes of 21 species including three species from Amaranthaceae as complex outgroup. Bootstrap values are based on 1000 replicates. The numbers on branches are bootstrap support values.

## Data Availability

The data that support the findings of this study are openly available in NCBI GenBank at https://www.ncbi.nlm.nih.gov under the accession no. MW192441. The associated BioProject, SRA, and Bio-Sample numbers of Illumina raw sequencing data of *Caroxylon passerinum* are PRJNA673337, SRS7622123, and SAMN16604536, respectively.
